# Cleaning-in-place of immunoaffinity resins monitored by in situ ATR-FTIR spectroscopy

**DOI:** 10.1007/s00216-015-8871-3

**Published:** 2015-07-10

**Authors:** Maxime Boulet-Audet, Bernadette Byrne, Sergei G. Kazarian

**Affiliations:** Department of Chemical Engineering, Imperial College London, South Kensington Campus, London, SW7 2AZ UK; Department of Life Sciences, Imperial College London, South Kensington Campus, London, SW7 2AZ UK

**Keywords:** Infrared spectroscopy, ATR FT-IR, Spectroscopic imaging, Immunoglobulin, Immunoaffinity chromatography, Unfolding

## Abstract

In the next 10 years, the pharmaceutical industry anticipates that revenue from biotherapeutics will overtake those generated from small drug molecules. Despite effectively treating a range of chronic and life-threatening diseases, the high cost of biotherapeutics limits their use. For biotherapeutic monoclonal antibodies (mAbs), an important production cost is the affinity resin used for protein capture. Cleaning-in-place (CIP) protocols aim to optimise the lifespan of the resin by slowing binding capacity decay. Binding assays can determine resin capacity from the mobile phase, but do not reveal the underlying causes of Protein A ligand degradation. The focus needs to be on the stationary phase to examine the effect of CIP on the resin. To directly determine both the local Protein A ligand concentration and conformation on two Protein A resins, we developed a method based on attenuated total reflection (ATR) Fourier transform infrared (FTIR) spectroscopy. ATR-FTIR spectroscopic imaging revealed that applying a carefully controlled load to agarose beads produces an even and reproducible contact with the internal reflection element. This allowed detection and quantification of the binding capacity of the stationary phase. ATR-FTIR spectroscopy also showed that Protein A proteolysis does not seem to occur under typical CIP conditions (below 1 M NaOH). However, our data revealed that concentrations of NaOH above 0.1 M cause significant changes in Protein A conformation. The addition of >0.4 M trehalose during CIP significantly reduced NaOH-induced ligand unfolding observed for one of the two Protein A resins tested. Such insights could help to optimise CIP protocols in order to extend resin lifetime and reduce mAb production costs.

**Electronic supplementary material:**

The online version of this article (doi:10.1007/s00216-015-8871-3) contains supplementary material, which is available to authorized users.

**Graphical Abstract:**
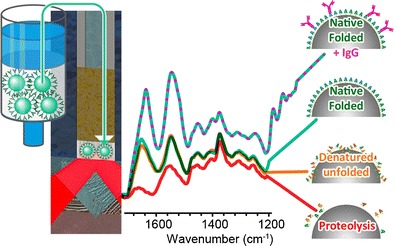
Experimental approach for analysing resin beads by attenduated total reflection alongside typical FTIR spectra

## Introduction

Biotherapeutics were worth over $165 billion in 2012 [1, 2] and accounted for 60 % of new drug patents filed by the ten largest pharmaceutical companies [3]. The market share of biotherapeutics is expected to grow to 32 % by 2023 [4]. Thanks to their remarkable specificity [5], many monoclonal antibodies (mAbs) have great potential for the treatment of chronic diseases, cancer and other life-threatening diseases [6]. Extracted from living systems cultured in highly controlled bioreactors, the largest industrial mAb batch yields are on the kilogramme scale while tonnes of synthetic drugs can be produced in a single batch [7, 8]. Because of the biological nature of mAb biotherapeutics, regulatory agencies demand more stringent clinical studies for biotherapeutics than for small molecule drugs due to the potential for adverse immune responses or organ-specific effects [2]. In addition, the requirements for high protein purity and low virus clearance values further increase the production cost. Despite the proven effectiveness of mAbs, the National Institute for Health and Clinical Excellence (NICE) often recommends against making them available to patients because of their prohibitive cost. NICE typically rejects mAbs costing more than £20 k per quality-adjusted life year (QALY) while some mAbs can cost more than £116 k per QALY [9]. Off-patent biosimilar mAbs should reduce the treatment price by 20–40 %, but this is a small reduction compared to the 80 % savings made for biosimilars of small molecule drugs.

With improved bioreactor efficiencies for mAb production, downstream processing now accounts for more than 70 % of total production costs. The immunoaffinity chromatography resin needed for mAb capture has the biggest impact on production, representing more than 50 % of the cost of goods [8]. Such resin comprises cross-linked agarose beads functionalised with Protein A which binds to the constant fragment crystallisable (Fc) of mAbs [10]. Protein A can be isolated from the native source, the cell wall of Gram-positive *Staphylococcus aureus*, but can also be expressed recombinantly in *Escherichia coli* [11, 12]. Protein A chromatography effectively removes ~98 % of the host cell proteins and other biological impurities from the culture fluid in a single step [13–15]. Unfortunately, Protein A chromatography suffers from a gradual loss of binding capacity over repeated purification cycles [16] as mAb aggregates and biological impurities can bind irreversibly to the column [16, 17].

Cleaning-in-place (CIP) protocols typically include washing the resin with alkaline solutions [18, 19, 13] to prevent contaminant build-up. Sodium hydroxide solutions can efficiently remove precipitated proteins, lipids and nucleic acids while inactivating bacteria, viruses, yeast and endotoxins [20, 21]. High pH conditions during CIP also inactivate microbes while removing contaminants that could carry over into subsequent purification cycles [22]. However, even with CIP, the mAb binding capacity of the Protein A resin binding capacity decays over purification runs, typically requiring replacement after 50 to 300 cycles [16, 18]. Replacing a single industrial-scale 1500-L protein A column can cost up to $12 M, not including the incurred production interruptions [8]. Important cost savings could result from optimisation of CIP protocols to extend the resin lifespan.

Previous reports point to ligand degradation during CIP as the primary factor causing binding capacity decay [23, 16]. A slight change in the protein conformation from alterations in the native tertiary structure contacts can even affect functionality [24]. GE Healthcare bioengineered the Protein A ligand for MabSelect SuRe^tm^, offering superior resistance to alkaline conditions. Substituting asparagine and glutamine residues greatly reduces protein sensitivity to deamidation, the removal of amide functions, at high pH [25]. Optimised CIP protocols using salt and excipients can help prevent ligand degradation during CIP [12].

Static and dynamic binding assays can quantify the binding capacity [17, 16, 26], but such analyses only provide information on the loss of function. Enzyme-linked immunosorbent assay (ELISA) can also be used to measure traces of Protein A ligand leaching from columns [13]; however, the amount of ligand lost cannot fully explain the loss of binding capacity. Without methods to reveal the underlying cause of Protein A ligand decay, efforts to optimise CIP protocols remain limited [17]. This study aimed to identify the main cause of Protein A ligand degradation and loss of binding capacity through direct analysis of the immobilised Protein A ligand.

By measuring the resin directly, attenuated total reflection (ATR) FTIR spectroscopy is particularly well suited to identify the cause of ligand degradation. Unlike other techniques such as circular dichroism, dynamic light scattering or mass spectrometry, FTIR spectroscopy and FTIR spectroscopic imaging can easily probe heterogeneous samples such as agarose beads. Previous studies have used thick transmission cells to study agarose beads in single layers [27]. However, the attenuated total reflection (ATR) mode avoids the issue of buffer from masking the protein’s spectral regions of interest. Because ATR mode probes only a thin layer of the sample adjacent to the surface, ATR-FTIR spectroscopy is much more sensitive to bound ligand than transmission mode. Infrared spectroscopy provides information on protein conformation [5, 28–33] and can thus determine the effect of CIP on the structure of the immobilised ligand.

We developed a highly reproducible ATR-FTIR spectroscopy method to probe agarose resin directly. Because the ATR mode requires an intimate contact between the sample and the internal reflection element, we applied pressure to the resin beads rather than decreasing their size [34], using polymer binders [35], or substituting the matrix for a flat surface [36, 37]. The absorbance measured from convex partials depends on the load applied [38]. Since a typical ATR accessory cannot control the load precisely enough for reproducible measurements, we mounted a load sensor to measure the force applied to the resin. This approach yielded highly reproducible spectra, revealing the Protein A density and conformation from only a few hundred resin beads without the need for extensive sample preparation [39].

After quantifying the static binding capacity from the mobile phase using 280 nm absorption, we analysed the stationary phase by ATR-FTIR spectroscopy. Resin samples were measured following a range of CIP conditions as well as during NaOH incubation in situ. This approach revealed the cause of binding capacity decay by determining the amount of adsorbed protein on the beads and the conformation of the immobilised protein ligand. Our results indicate the minimum NaOH concentration inducing ligand unfolding. A disaccharide was also found to reduce the impact of alkaline conditions. This approach and derived insights should help direct optimisation of CIP protocols to preserve the resin’s binding capacity.

## Materials and methods

### Monoclonal antibody preparation

The monoclonal antibody (mAb) expression and purification is described in detail elsewhere [31]. Glutamine Synthetase Chinese Hamster Ovary (GS-CHO) cell lines that express the chimeric B72.3 immunoglobulin G gamma 4 (IgG4) were provided by Lonza (Lonza Biologics, Basel, Switzerland). The mAb concentration in the different media samples was quantified by ELISA (Montgomery, TX, US) and UV–vis spectroscopy using a Nanodrop Lite system (Thermo, Wilmington, DE, USA). The media containing the protein was then desalted using a HiPrep 26/10 desalting column (GE Healthcare Life Sciences) equilibrated with Buffer A (20 mM phosphate, pH 7.4). Subsequently, the IgG4 mAbs were captured using protein A chromatography with Recombinant Protein A Sepharose® FF resin (Generon Ltd, UK). The protein was further purified by anion exchange chromatography using a HiTrap Q FF prepacked with Q Sepharose (GE Healthcare Life Sciences). The purified protein was then concentrated by centrifugal filtration using a molecular weight cut-off of 50 kDa to 4 mg/mL for the stock solution.

### Resin preparation and static binding capacity assays

Pierce Control Agarose Resin (Thermo Scientific, USA), Recombinant Protein A Sepharose® FF resin (Generon Ltd, UK) and MabSelect™ SuRe™ Protein A resin (GE Life Sciences, UK) are constituted of porous cross-linked agarose beads ranging from 20 to 160 μm in diameter. For in situ measurements, resin samples were mixed with a NaOH solution to achieve a 420 mM final concentration just before ATR-FTIR spectroscopic kinetic measurements.

**Fig. 1:**
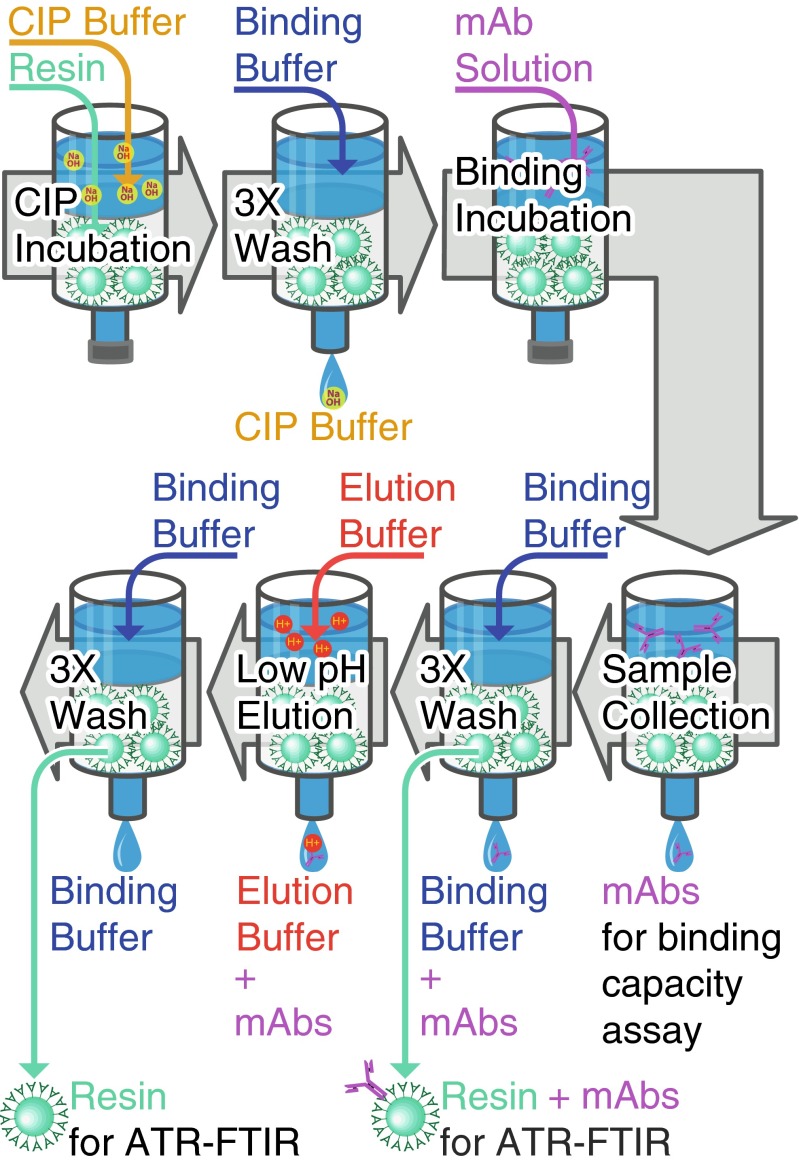
Flow chart depicting the method for the preparation of the resin samples

To increase throughput and test multiple CIP conditions, ATR-FTIR spectroscopic measurements were performed after the incubation (ex situ) following the flow chart in Fig. 1. Protein A Sepharose, MabSelect SuRe and 10 μL of sedimented resin were transferred into 1-mL micro spin columns (Bio-Rad, USA). To test the effect of alkalinity, resin samples were subsequently incubated for 10 h with the cleaning-in-place solution containing NaOH at concentrations ranging from 0 to 6450 mM. To enhance diffusion, samples were stirred gently on a roller during incubation. Protein A resin is typically sanitised with 3 CV of NaOH at 0.1 to 1 M, with a 30-min contact time [16], corresponding to 20 sanitation cycles. To test the effect of d-(+)-trehalose (Sigma Aldrich, USA) on Protein A ligand during CIP, a series of resin samples were incubated for 16 h in 215 mM NaOH mixed with disaccharide at concentrations ranging from 0 to 700 mM. Following CIP, resin samples were washed three times with 20 mM phosphate buffer at pH 7.4 by spinning the column. A mAb stock solution was then added to obtain a final concentration of 4.55 mg/mL in 0.2 mL of binding buffer. After a 3-h contact time, the concentration of mAbs left in solution was measured using UV at 280 nm with *E*^1%^ = 13.7. The static binding capacity *q* was calculated from the difference in protein concentration measured. To remove unbound mAbs, samples were subsequently washed with binding buffer three times. Aliquots of resin with bound mAbs were then collected to determine the binding capacity by probing the stationary phase with ATR-FTIR spectroscopy. To release and elute the mAbs from the resin, samples were subsequently washed with 20 mM phosphate buffer pH 3.0. The mAb-free resin samples were washed again with binding buffer three times before performing another series of ATR-FTIR spectroscopic measurements.

### ATR-FTIR experimental set-up

**Fig. 2:**
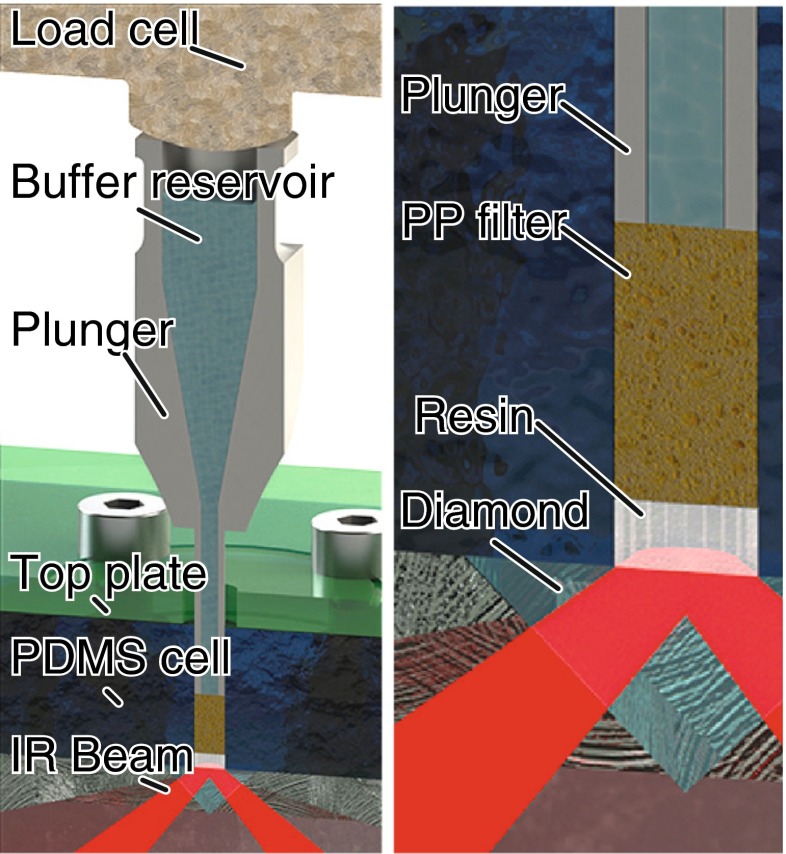
Schematic presentation of the experimental setup used for resin analysis by ATR-FTIR spectroscopy and spectroscopic imaging. The polydimethylsiloxane (PDMS) cell was clamped to the ATR accessory using an acrylic top plate while a plunger applied the load on the resin beads. The force applied was measured by a load cell mounted on top of the plunger

The sample cell shown in Fig. 2 was made from Sylgard® 184 PDMS (Dow Corning Corporation, USA) cast from an acrylic 3D printed mould (Shapeways, New York, USA). This was attached to a Golden Gate (Specac, UK) diamond attenuated total reflection accessory to probe the bottom of the cell. Two microlitres of sedimented resin beads were first loaded into the 1.3-mm well and gravity packed onto the diamond internal reflection element (IRE) before collecting the buffer background spectrum. To obtain a good contact between the beads and the IRE, a stainless steel plunger pressed the resin with a porous polypropylene (PP) filter. The excess buffer flowed through the PP into the plunger’s buffer reservoir. The PP filter distributed the load evenly while allowing the flow of buffer to prevent trapping air in the well. An iLoad Mini load cell (Loadstar Sensors, USA) monitored compression force as the plunger was lowered down the well at ~0.5 mm/min. Once the filter touched the resin beads, the force reading rose rapidly by around 30 % as the PP filter deformed under load. Unless specified otherwise, target load after relaxation was 200 g (1.5 MPa). Typical compression curves with and without resin are shown in Electronic Supplementary Material (ESM) Fig. S1. Under load, the beads flatten sufficiently to be probed effectively by the evanescent wave.

After collecting spectra, the entire PDMS cell was unclamped to clean the IRE and the PDMS cell. Recovered beads appeared unaffected by the gentle load as shown in ESM Fig. S2. The sample loading, cell cleaning, spectrum and background collection took less than 6 min per aliquot with five aliquots measured for each condition tested.

The concentration of isolated Protein A from *S. aureus* standard solution series was determined by UV absorption at 280 nm with a Nano drop Lite (Thermo, USA) using *E*^1%^ = 1.65 [10]. The absorbance of the amide II was integrated to quantify the protein A concentration using the calibration curve (ESM Fig. S3).

### ATR-FTIR spectroscopy

Attenuated total reflection (ATR) Fourier transform infrared (FTIR) spectra were collected using a Varian 670 spectrometer (Agilent Technologies, UK) equipped with a Peltier-cooled deuterium tri-glycine sulphate (DTGS), single-element pyroelectric detector. Hydrated beads in buffer were probed by ATR mode using a reflection diamond Golden Gate accessory with optics suitable for spectroscopic imaging (Specac, UK) [40]. The 45° angle of incidence resulted in the depth of penetration of~1.2 μm at 1600 cm^−1^ on the diamond’s surface [40, 41]. ATR-FTIR spectra were collected at a 4-cm^−1^ resolution using an aperture equivalent to a resolution of 0.5 cm^−1^. For ex situ measurements after incubation, 64 scans were co-averaged while 128 scans were collected for each time frame in situ experiments during incubation.

For ATR-FTIR spectroscopy using a single-element DTGS detector, infrared spectra were collected using Resolution Pro 5.2 (Agilent Technologies Ltd, USA) before being imported into MATLAB. We estimated that the optimal subtraction coefficient for the buffer was around 0.95 based on the subtraction of the combination band (libration + OH bending) at 2125 cm^−1^ [42]. Despite a ~5 % water buffer over subtraction, we fixed the subtraction coefficient to 1 to improve the measurement reproducibility. The dependence of the depth of penetration on the wavelength was also corrected [43].

### ATR-FTIR spectroscopic imaging

For ATR-FTIR spectroscopic imaging, the diamond ATR accessory was placed in an external large sample compartment and data collected using a cooled mercury-cadmium-telluride (MCT) focal plane array (FPA) displaying 128 by 128 elements capable of collecting 16384 spectra simultaneously [32]. Since about a quarter of the FPA was illuminated by the optics, the size of the images was set to 64 by 64 (4096 spectra). The imaging spectra were also collected at 4 cm^−1^ and 64 scans were co-averaged for each image. The focusing lenses of the imaging IR accessory corrected partially for the beam aspect ratio distortion, resulting in a near square field of view of ~0.64 by ~0.52 mm [32].

Interferograms were collected using Resolution Pro 5.2 and saved as binary files. All subsequent data analysis operations were performed by a MATLAB (MathWorks, Natick, MA, USA) custom code as described elsewhere [31]. The algorithm applied an offset using the 1900 to 1850 cm^−1^ window before correcting for the penetration depth variation depending on the wavelength of light [43].

## Results and discussion

### Effect of load on resin by ATR-FTIR spectroscopic imaging

To better understand the causes of binding capacity decay, the Protein A resin needs to be probed directly. ATR-FTIR spectroscopy is well suited for probing solid materials while being sensitive to protein conformation [28, 29]. However, the ATR evanescent wave only probes a thin sample layer in close contact with the IRE as shown in Fig. 2. Measuring chromatography resins by ATR-FTIR spectroscopy thus poses a challenge as they typically consist of convex 30 to 150 μm porous agarose microspheres. Compared to a thick liquid film, an area covered by 4-μm beads would absorb infrared light five times less while 50-μm beads would result in only 2 % of the potential absorbance [38]. Reducing bead size would thus increase sensitivity [34], but would not represent the industry’s standard chromatographic media. Instead, we opted for squeezing beads onto the internal reflection element in order to increase contact [38].

With a Young’s modulus of only 100 to 200 kPa [44], the contact of soft agarose beads with the IRE should depend on the load [38]. Mounted on the plunger (Fig. 2), a load cell accurately measured the force on the resin beads (±30 mN). After lowering the plunger, the stress reduces as the filter relaxes (ESM Fig. S1). To visualise the contact between the resin beads and the IRE, a focal plane array (FPA) detector collected ATR-FTIR spectroscopic images of the resin beads under load.

**Fig. 3:**
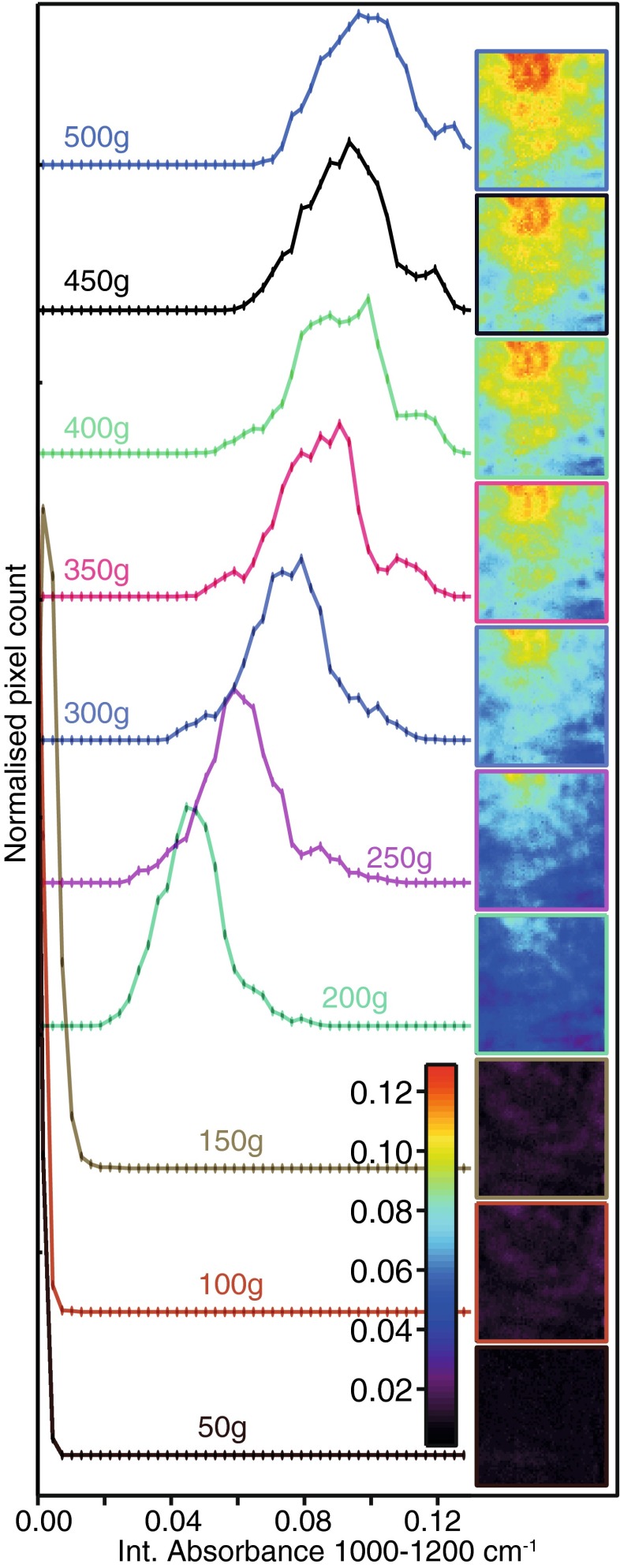
ATR-FTIR spectroscopic images (*right*) based on the distribution of integrated absorbance in the 1200 to 1000 cm^−1^ region for Protein A Sepharose resin under a 50- to 500-g load. The corresponding histograms (*left*) show the pixel count rate as a function of the integrated absorbance. The images’ field of view was ~0.64 × 0.52 mm [32]

Figure 3 shows that the contact between the beads and the IRE varies with the applied load. Integrating absorbance of the main polysaccharide band (1200–1000 cm^−1^) generated spectroscopic images of the agarose resin beads. A load below 150 g did not overcome the friction between the PP filter and the PDMS well, resulting in blank images. Over 200 g, the PP filter moved to the bottom of the well and pressed the resin beads against the internal reflection element, resulting in substantial absorption of the evanescent wave. The histogram of the image at 200 g corresponds to a symmetrical and relatively narrow absorbance distribution, centred around 0.045 absorbance units. Under heavier load, resin beads deform further leading to a distribution maximum shifting towards higher absorbance values as contact improves. Simultaneously, the absorbance distribution widened as a result of more heterogeneous images. Upon high pressure, the apparent diameter of squashed microspheres can become even greater than their uncompressed diameter [38]. Despite weaker absorbance, we choose to apply the minimum load required to obtain a sufficient and reproducible contact (200 g or 1.5 MPa). The resin polysaccharide absorbance vanished after removing the load as beads relaxed to their original spherical shape. Unlike other studies that relied on spectra collected from dried and crushed resin [39], this approach does not appear to damage agarose beads (ESM Fig. S2).

Multiple load cells to ensure reproducible contact could be used in combination with FTIR spectroscopic imaging to allow high-throughput measurements [31, 32, 45]. FTIR spectroscopic imaging could also prove particularly powerful to investigate the distribution of impurities building up in flowing systems. Since beads cover the field of view evenly under a 200-g load, FTIR spectroscopic experiments may also be conducted using conventional single-element detectors. Measuring agarose beads with conventional FTIR spectroscopy could thus become a routine method to support protocol validation.

### ATR-FTIR spectra of resin

Since infrared spectra are indicative of the particular chemical composition, we used our resin probing platform to compare three types of hydrated chromatographic resins. This method produced a high signal-to-noise ratio and reproducible ATR-FTIR spectra from only a few hundred beads and in less than 6 min per aliquot.

**Fig. 4:**
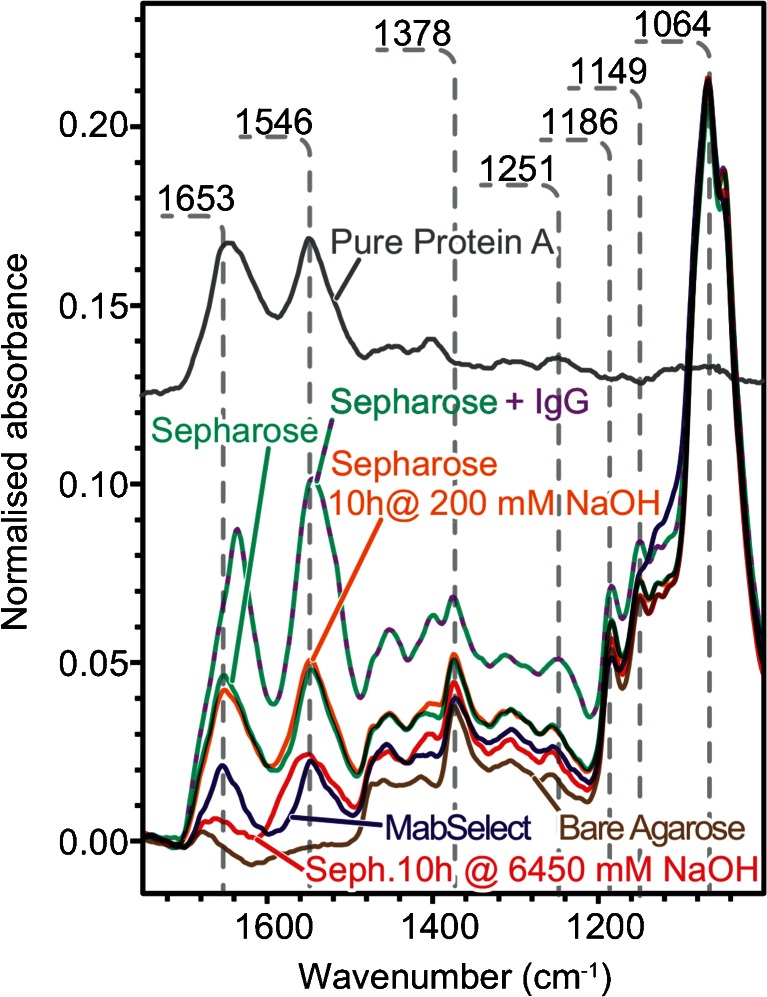
ATR-FTIR spectra of untreated agarose resin (*brown*) under a 200-g load, MabSelect Sure resin (*blue*), Sepharose resin (*teal*), Sepharose resin with bound mAbs (*teal with fuchsia dashes*), Sepharose resin treated for 10 h with 200 mM NaOH (*orange*) and 6450 NaOH (*red*). The ATR-FTIR spectrum of pure Protein A in solution (*grey*) is offset to improve visualisation

Figure 4 shows clear differences between bare agarose, rProtein A Sepharose and MabSelect resins. The brown spectrum of bare agarose shows strong bands at 1064 cm^−1^ due to C–O stretching/C–O–H bending from hydroxyl functions and at 1378, 1186 and 1149 cm^−1^ from polysaccharide skeletal modes [46, 47]. No bands appear between 1500 and 1700 cm^−1^, but the slightly negative absorbance results from over-subtracting the absorbance of the band corresponding to the bending mode of water at 1638 cm^−1^ from the displaced buffer [48]. The spectrum of agarose functionalised with Protein A (Sepharose, teal curve) presents strong protein bands. As a reference, the grey trace in Fig. 4 shows the infrared absorption of Protein A with the expected amide bands peaking at 1653, 1546 and 1251 cm^−1^. The maximum amide I absorption at 1653 cm^−1^ indicates a predominantly helical conformation [28, 29], in agreement with the known crystalline structure [49]. Polysaccharide peaks overlap protein side chain vibration modes between 1500 and 1300 cm^−1^ and the amide III band [30], but, fortunately, do not interfere with the amide I and II bands above 1500 cm^−1^. Presented as an indigo curve, the infrared spectrum of MabSelect resin shows some slight differences in the polysaccharide region around 1149 cm^−1^ compared to the rProtein A Sepharose resin. The difference might arise from the higher degree of cross-linking for MabSelect matrix [12]. However, MabSelect has substantially weaker amide I and II bands, implying a lower Protein A ligand density. Using this method, we could thus probe immobilised Protein A ligand directly on agarose beads using ATR-FTIR spectroscopy for the first time.

### Protein A ligand density quantification

ATR-FTIR spectroscopy represents a convenient and non-destructive method to quantify Protein A ligand density on resin beads. Ligand density is typically estimated roughly by UV absorption difference in the buffer. Alternatively, concentration can be measured in transmission mode through thin resin samples after adjusting the refractive index, which is not very practical [14]. Elemental or amino acid analysis represents another alternative by accurately quantifying ligand density, but such techniques require matrix digestion [50]. In transmission mode, infrared absorption would saturate in the amide I and II regions when using a beam path thicker than 10 μm, considerably thinner than the smaller agarose beads studies (>50 μm). Because the evanescent wave probes around 1 μm deep into samples when using a diamond with a 45° angle of incidence [41, 43], spectra collected in ATR mode do not saturate in the mid-infrared region.

Since the absorbance of the amide bands is proportional to the protein concentration, infrared spectra can thus be used to determine the amount of bound Protein A ligand. Using a protein A calibration curve, we calculated a local concentration of 84 ± 9 mg/mL for rProtein A Sepharose, more than twice the amount on MabSelect (31 ± 9 mg/mL). We expected a difference since manufacturers reported a Protein A ligand density of 3.5 and 1.64 mg/mL for Sepharose and MabSelect, respectively [12]. Nevertheless, comparable binding capacities were still reported for MabSelect [16, 12]. The shallow penetration depth of the proving wave explains the proportionally high protein absorbance measured relative to the protein to dry polysaccharide ratio (~12 % = 3.5/29 mg). ATR is thus more sensitive to bound Protein A ligands on the surface than in the agarose matrix [14]. Compared to transmission, ATR mode is thus a more sensitive method for the quantification of the Protein A ligands. ATR spectra are indicative of the local protein density in a layer adjacent to the surface of internal reflection element rather than of the total amount of Protein A ligand. The local concentration should still be proportional to the total density when assuming an even coverage.

### In situ cleaning-in-place

The ligand must be measured directly rather than indirectly through reduced binding capacity to identify the cause of binding capacity. Because binding capacity loss occurs without product following CIP, alkaline conditions appear to be mainly responsible for Protein A ligand degradation [16, 12, 26]. Under chemical stress such as high pH, proteins will eventually denature as a result of proteolysis, deamidation or unfolding. Proteolysis can cause loss of the entire protein ligand or protein fragments from the resin, decreasing ligand density. Under denaturing conditions, the mobility of the backbone increases which in turn allows the protein conformation to change [51]. Deamidation also occurs faster at high pH causing the asparagine residues to react with the backbone, resulting in side chain alterations and changes in protein conformation. Because binding capacity cannot discriminate between these effects, the ligand must be analysed directly instead.

Indicative of the Protein A conformation, ATR-FTIR spectroscopy revealed that protein unfolding is the likely cause of Protein A ligand denaturation (<1 M NaOH). Proteolysis, unfolding and deamidation are detectable in the amide band regions [30, 31, 52–54]. The orange spectrum of Fig. 4 shows that exposure to 200 mM NaOH for 10 h affects mainly the amide I and II band positions. Such a band shift can be indicative of conformational change. Since the amide band absorbance remained constant after CIP and the wash step, our result implies that proteolysis is not an important contributor to ligand degradation. In addition, no COOH peak appeared between 1710 and 1760 cm^−1^ [30], suggesting that few Asn side chains underwent deamidation. Based on infrared spectra, Protein A unfolding is thus the most likely cause of ligand denaturation under typical CIP alkaline conditions.

Since the amide II band responds more to conformation changes than the amide I [53, 29], we calculated the centre of gravity (COG) of the 1575–1525 cm^−1^ amide II region precisely to detect the effect of NaOH on the protein ligand. The COG does not depend on the absolute absorbance and can detect band shifts smaller than the spectral resolution [52, 55]. In a sealed system, we incubated Protein A resin samples under typical NaOH concentrations used for CIP while collecting infrared spectra to calculate the amide II COG.

**Fig. 5:**
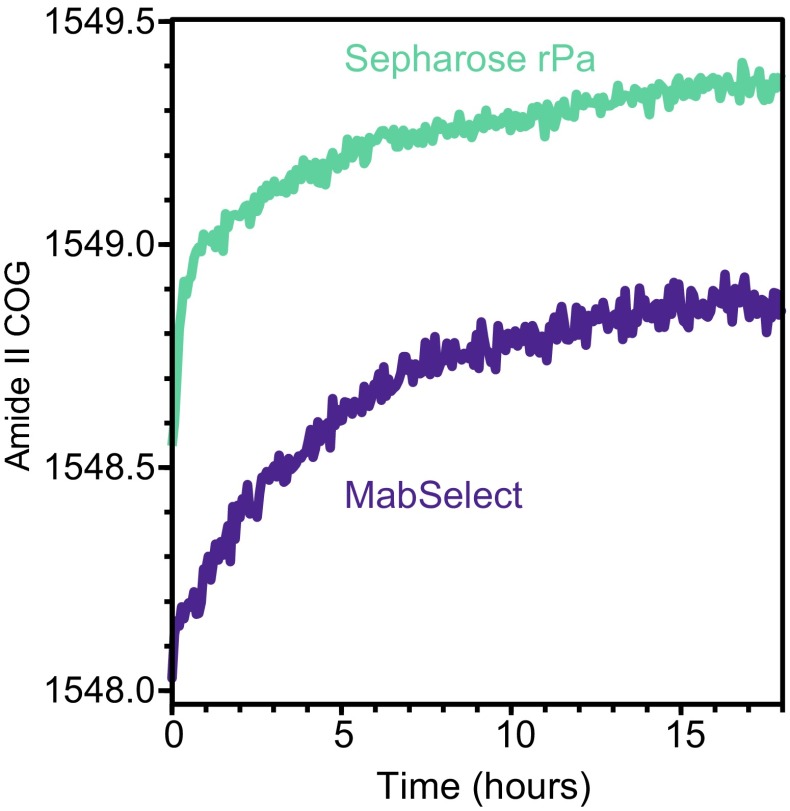
Amide II band centre of gravity (COG) as a function of time for a typical rProtein A Sepharose resin and MabSelect resin under 420 mM NaOH CIP. See ESM Fig. S4 for a close-up of the amide I and II region for different samples under a 200-g load

Figure 5 shows that ATR-FTIR spectroscopy can detect subtle Protein A ligand conformation changes during CIP in situ. For both rProtein A Sepharose and MabSelect resin exposed to 420 mM NaOH, the amide II COG shifted to a higher wavenumber as a function of time. The COG shifted faster at the beginning of the incubation than after ~4 h, particularly for the Sepharose resin. The amide II band still continued to rise, albeit slowly, after exposing the resin to NaOH for 18 h suggesting that unfolding is not complete even after prolonged exposure times. Under such CIP conditions, the resin would be expected to have lost substantial binding capacity and be rendered unusable [16]. ATR-FTIR spectroscopy could thus follow the ligand’s structure during CIP steps without the need to interfere with the production pipeline or rely on mAb binding interactions.

The varying rate of amide II COG shift could be attributable to buffer diffusion as the thin layer near the bead surface was exposed to NaOH first before it could have diffused through the matrix. According to this hypothesis, less frequent but longer exposure periods could be less detrimental to the ligand protein conformation while being equally effective at CIP. To validate such a hypothesis, a subsequent study would be needed, where the buffer conditions are changed sequentially.

### Effect of NaOH concentration on the binding capacity and resin ligand

In addition to the exposure time, the concentration of NaOH was also identified as an important parameter for CIP protocols [16]. Because only one sample could be measured in situ, testing different conditions would be very time consuming. Instead, we incubated several individual resin samples under a range of NaOH concentrations simultaneously, then washed them with binding buffer prior to static binding capacity assays using stock mAb solution. Subsequently, half of the resin aliquots were washed with low pH elution buffer to release the mAbs. Resin samples with and without bound mAbs were then analysed by ATR-FTIR spectroscopy to quantify the adsorbed protein density using the amide II integrated absorbance against a calibration curve (ESM Fig. S3). With this approach, many resin aliquots could thus be studied rapidly to test a wide range of buffer compositions.

**Fig. 6:**
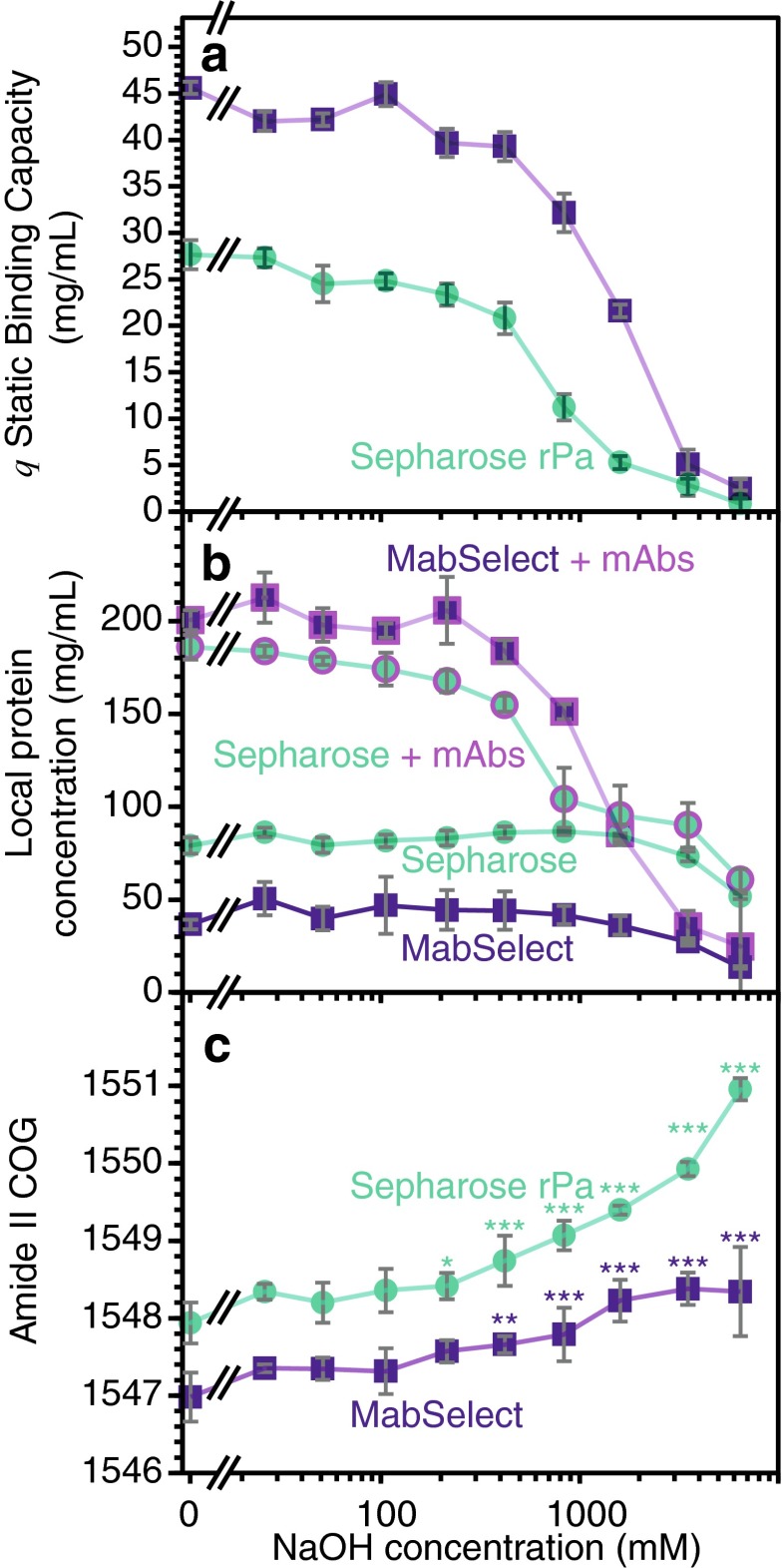
**a** Static binding capacity measured from the mobile phase. **b** Local protein concentration calculated from resin ATR-FTIR spectra of resin with mAbs (*fuchsia marker outline*) and without bound mAbs (no contour). **c** Amide II centre of gravity (COG) measurements for rProtein A Sepharose resin (*teal*) and MabSelect SuRe resin (*indigo*) following a 10-h incubation with a range of NaOH concentrations after low pH elution. The *error bars* represent the standard deviation; *p < 0.05, **p < 0.01, and ***p < 0.001 as assessed by one-way ANOVA

As previously reported [16], NaOH can substantially reduce the static binding capacity (*q*) of protein A resin, although less for the more alkaline resistant MabSelect SuRe. Figure 6a shows the decrease in *q* of Sepharose and MabSelect SuRe resin as a function of the NaOH concentration for a contact time of 10 h, equivalent to 20 CIP cycles. For the same amount of protein A resin, MabSelect appears to have a greater *q* than Sepharose at 0 mM NaOH. For both types of resin, the binding capacity decreased slowly with increasing NaOH concentration. CIP with more than 800 mM NaOH severely decreases the binding capacity for both types of resin. At 1600 mM NaOH, however, MabSelect SuRe preserved half of its binding capacity while Sepharose nearly did not bind any mAbs. At even higher NaOH concentration, both resins lost almost all binding capacity. To determine the underlying cause of binding capacity loss, resin samples with and without bound mAbs were analysed directly by ATR-FTIR spectroscopy.

Proteolysis occurs at high NaOH concentrations for both types of resin, but not significantly under typical CIP conditions. The drop in amide band absorption indicated by the red trace in Fig. 4 shows that incubating rProtein A Sepharose with 6450 mM NaOH causes ligand proteolysis. Figure 6b shows the local protein concentration calculated from the bead spectra incubated under a range of NaOH conditions and washed with elution buffer to unbind mAbs. Compared to the control, only the 6450 mM NaOH concentration measured caused a significant drop in Protein A concentration for both rProtein A Sepharose and MabSelect. Since typical CIP protocols do not expose the resin to such harsh conditions [19, 16], this result confirms that binding capacity decay does not arise from proteolysis. Hence, measuring Protein A ligand leaching by ELISA [13] is unlikely to be indicative of binding capacity decay reasserting the need for direct resin measurements.

The amount of adsorbed protein detected by ATR-FTIR spectroscopy also depends on the CIP conditions and the type of resin. When incubating the protein A resin with mAbs, the absorbance of the amide bands increase due to the adsorbed protein (teal with fuchsia dashes, Fig. 4). Figure 6b shows the difference between the local protein concentration between resin washed with elution buffer (Protein A only) and washed with binding buffer (Protein A + mAbs, fuchsia marker outline). This difference vanishes above 1600 mM NaOH concentration as a non-significant amount of mAbs is adsorbed to the resin beads. Once again, MabSelect SuRe appears to better preserve binding capacity at 800 mM NaOH with a significant difference of protein adsorbed compared to the sample without bound mAbs.

Without bound mAbs, NaOH affects the protein conformation of the protein A ligand under typical CIP conditions, particularly for the less resistant rProtein A Sepharose. From the same spectra set, Fig. 6c shows the amide II COG of the resin aliquots washed with elution buffer. Different ligand density could explain the difference in COG between Sepharose and MabSelect at 0 mM NaOH. The lower Protein A ligand density on MabSelect SuRe caused larger measurement standard deviations. For both resin types studied, a definite trend arises as the amide II COG shifted toward higher wavenumbers with increasing NaOH concentration. Treatment with only 200 mM NaOH resulted in a significant amide II band COG shift for rProtein A Sepharose while a significant change for MabSelect was only observed at concentrations of NaOH above 400 mM. The superior resistance of MabSelect becomes even more apparent at high concentration with the Amide II band of Sepharose resin shifting much more at 3510 mM NaOH. Engineered to improve high pH stability by replacing asparagine residues [12, 56, 16], the greater stability of MabSelect was expected. Despite the larger statistical errors, our results suggest that MabSelect is indeed more resistant to protein unfolding under typical conditions used for CIP.

**Fig. 7:**
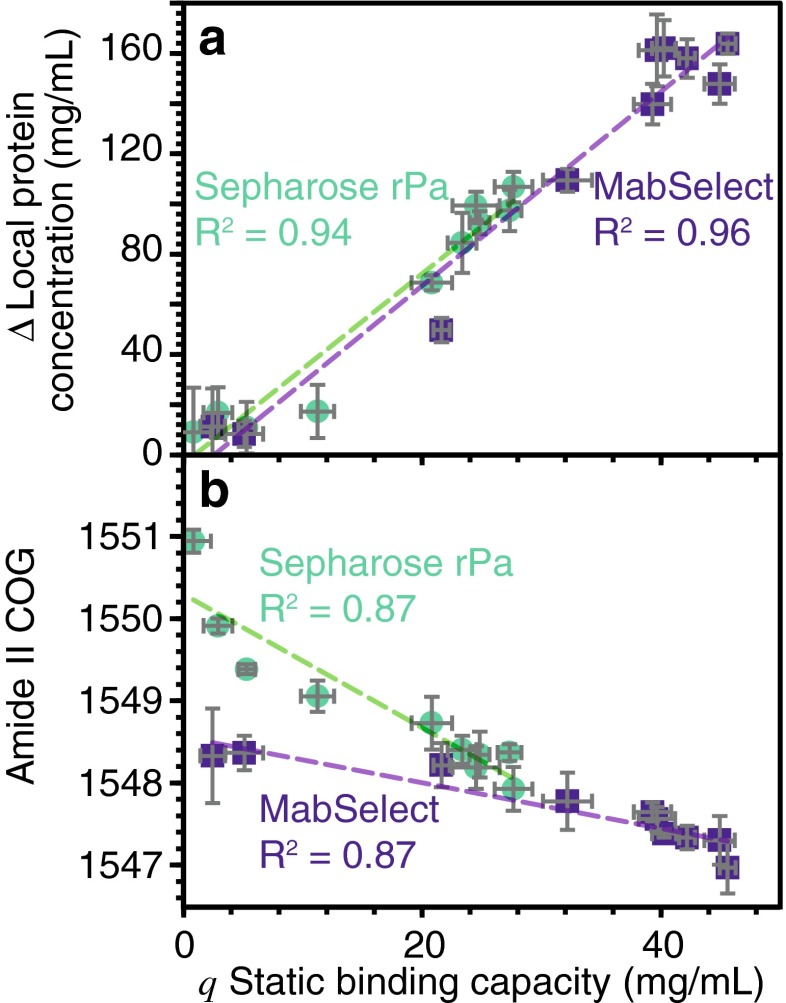
**a** Difference in local protein concentration measured by ATR-FTIR spectroscopy as a function of the static binding capacity (*q*). **b** Amide II band centre of gravity (COG) as a function of the static binding capacity

The local protein concentration on the beads, measured by ATR-FTIR spectroscopy, relates to the static binding capacity (*q*) measured by 280 nm. Subtracting the local protein concentration of the resin without mAbs removes the contribution of the adsorbed Protein A ligand. Figure 7a shows the difference in adsorbed protein concentration as a function of *q*. The amount of adsorbed mAbs detected by ATR-FTIR varies linearly with the concentration of mAbs measured in the mobile phase (*R*^2^ = 0.94 for Sepharose and *R*^2^ = 0.96 for MabSelect). As expected, the same linear trend was observed for both types of resin since the same mAb was used. We concluded that measuring the stationary phase by ATR-FTIR to quantify the ligand density could thus present an alternative to UV 280 nm for the quantification of the static binding capacity. Under milder conditions, more subtle conformation changes such as altered tertiary contacts can still affect binding functionality in the absence of proteolysis. With a high degree of interplay expected between tertiary and secondary structural changes [24], we evaluated the potential of infrared spectra as a proxy for binding capacity.

The Protein A ligand amide II band measured by ATR-FTIR spectroscopy without bound mAbs also appears to relate to *q*. Figure 7b shows the amide II band COG as a function of *q* for both Sepharose and MabSelect SuRe. For both types of resin, the conformation-dependent amide II COG correlates with *q* (*R*^2^ = 0.87 for Sepharose and MabSelect). The higher ligand density of Sepharose could explain the steeper negative trend compared to MabSelect as more protein could unfold. Hence, this result suggests that the protein conformation change could relate to the decay of the binding capacity. The amide II COG has the benefit of allowing in situ measurements without relying on mAb binding interactions.

**Fig. 8:**
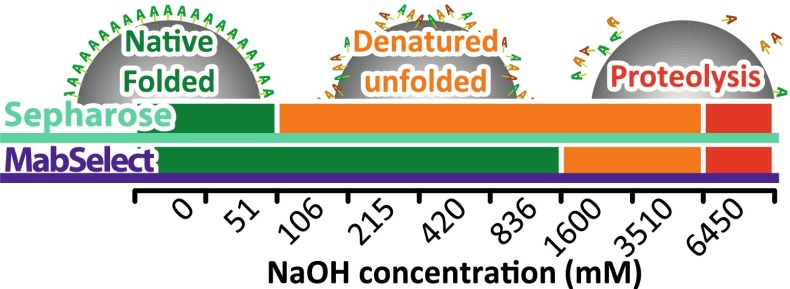
Schematic illustrating the range of NaOH concentrations that induced unfolding and proteolysis for the Sepharose and MabSelect resins

The schematic in Fig. 8 illustrates the distinction between denaturation caused by proteolysis (red) and through protein unfolding (orange). Our results suggest that only unfolding occurs at NaOH concentrations typically used for CIP (<1000 mM). Avoiding or reducing conditions causing ligand unfolding should thus help preserve binding capacity and extend resin lifespan. Since the resin binding capacity decays over repeated CIP cycles, the exposure time to NaOH should directly relate to ligand degradation. Since substantial binding capacity loss occurs under such conditions, we hypothesised that ATR-FTIR spectroscopy could provide information on resin binding capacity. To monitor ligand degradation in situ, such ATR-FTIR spectroscopic study of resins should represent an alternative to static and dynamic binding assays requiring mAbs.

### Effect of trehalose with NaOH on the resin

In addition to reducing NaOH concentration and exposure time, xylitol, ethylene glycol and NaCl have previously been found to help preserve resin binding capacity [16]. It is possible that such excipients would offer some degree of protection from denaturation. Some saccharides are known to be able to protect bound water and form hydrogen bonds, which help preserve protein conformation [57–60]. Since trehalose is known to stabilise proteins [61, 62], we investigated the effect of NaOH on the two resin types in the presence of a range of disaccharide concentrations.

**Fig. 9:**
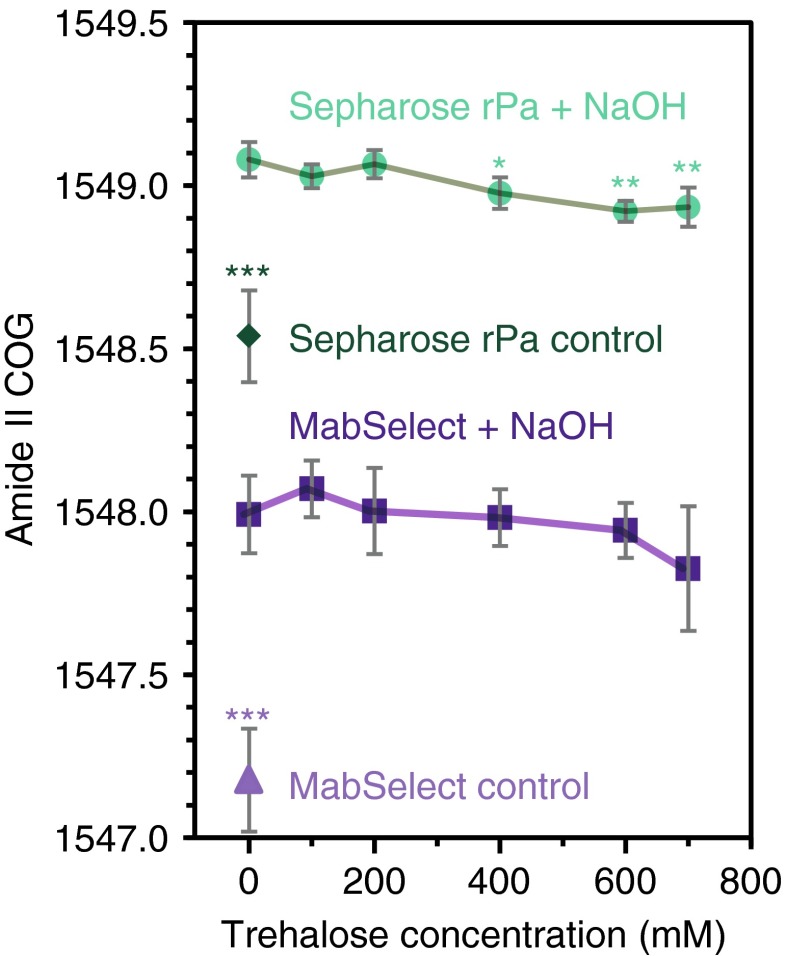
Amide II band centre of gravity (COG) as a function of NaOH and trehalose concentration for Sepharose (*teal*) and MabSelect (*indigo*) after a 16-h incubation period. The *error bars* represent the standard deviation; *p < 0.05 and **p < 0.01 as assessed by one-way ANOVA

Figure 9 shows a clear difference between the amide II COG for the control samples and those incubated for 16 h with 215 mM NaOH. In addition, rProtein A Sepharose was significantly more affected by the NaOH, with a COG shift of 0.6 ± 0.1 cm^−1^, than MabSelect resin, with a COG shift of 0.4 ± 0.1 cm^−1^. For rProtein A Sepharose, >400 mM trehalose significantly reduced, but did not abolish, the NaOH-induced shift. The trehalose may help to reduce the denaturing effect of NaOH by stabilising the Protein A ligand. Higher trehalose concentration may have a greater stabilisation effect, but buffer dilution and the 1.8 M solubility limit the maximum concentration in solution during CIP [63]. However, addition of up to 700 mM trehalose to the buffer did not cause any significant reduction of the amide II COG for MabSelect SuRe resin. These results may be due to the greater resistance of MabSelect SuRe to NaOH. Alternatively, the larger errors resulting from the lower ligand density could have hidden the effect of trehalose. Using our experimental approach, many more conditions could be rapidly screened to directly evaluate the effect of the buffer alkalinity on the Protein A ligand’s conformation either after CIP or in situ.

## Conclusions

This work represents the first studies of protein ligand denaturation on agarose stationary phase by infrared spectroscopy. By probing only a thin layer near the resin surface, ATR-FTIR spectroscopy proved particularly well suited to study Protein A ligand immobilised on soft agarose matrix. ATR-FTIR spectroscopic imaging confirmed that a homogenous contact between the IRE and resin beads can be obtained by applying a well-controlled load. If the load could be controlled separately in multiple wells, spectroscopic imaging could also allow high-throughput studies of multiple conditions simultaneously [45, 31]. ATR-FTIR spectroscopy allowed measurement of both ligand conformation and the amount of adsorbed protein on the beads following cleaning-in-place (CIP). Because proteolytic degradation of the Protein A ligand reduces IgG binding, the local protein concentration correlates with the static binding capacity assay results. Under milder conditions, cooperative changes in the tertiary and secondary structure conformations can still affect ligand functionality [24]. Our approach revealed that NaOH induced a conformational change in the protein ligand and that the binding capacity correlates with infrared absorption frequencies.

At NaOH concentrations typically used for CIP, between 100 and 1000 mM, ATR-FTIR spectroscopy detected significant conformational changes, but no significant ligand loss. We concluded that binding capacity decay following a typical CIP protocol result from Protein A ligand denaturation due to unfolding rather than proteolysis.

The results also revealed that high concentration trehalose solutions can reduce the effect of NaOH on Protein A conformation for rProtein A Sepharose. Since trehalose is mass-produced for sweetening applications [63], adding the disaccharide to CIP protocol represents an economical solution to extending immunoaffinity resin lifespan.

Unlike UV spectroscopy [16], nearly every organic molecule absorbs mid-infrared light, resulting in characteristic spectra with multiple distinct peaks [30, 32] allowing identification of host cell proteins, aggregated mAbs, nucleic acids, fatty acids, phospholipids, cholesterol or any other contaminants building up during purification. Ultimately, ATR spectroscopic sensors could be embedded to column casings to monitor the build-up of contaminants as well as Protein A ligand degradation in situ. By providing direct insights into the state of the protein A ligand, stationary phase sensing could thus deliver important improvements to the downstream purification process.

## Electronic supplementary material

ESM 1 (PDF 1.04 mb)
