# Supplementary material for: Cleaning-in-place of immunoaffinity resins monitored by in situ ATR-FTIR spectroscopy
Source: Anal Bioanal Chem. 2015 Jul 10;407(23):7111–22. doi: 10.1007/s00216-015-8871-3 (PMC4551555; doi:10.1007/s00216-015-8871-3)
Supplement: Supplementary file 1 — (PDF 1.04 mb) [file 216_2015_8871_MOESM1_ESM.pdf]

Analytical and Bioanalytical Chemistry

Electronic Supplementary Material

Cleaning-in-place of immunoaffinity resins monitored by *in situ* ATR-FTIR spectroscopy

Maxime Boulet-Audet, Bernadette Byrne, Sergei G. Kazarian

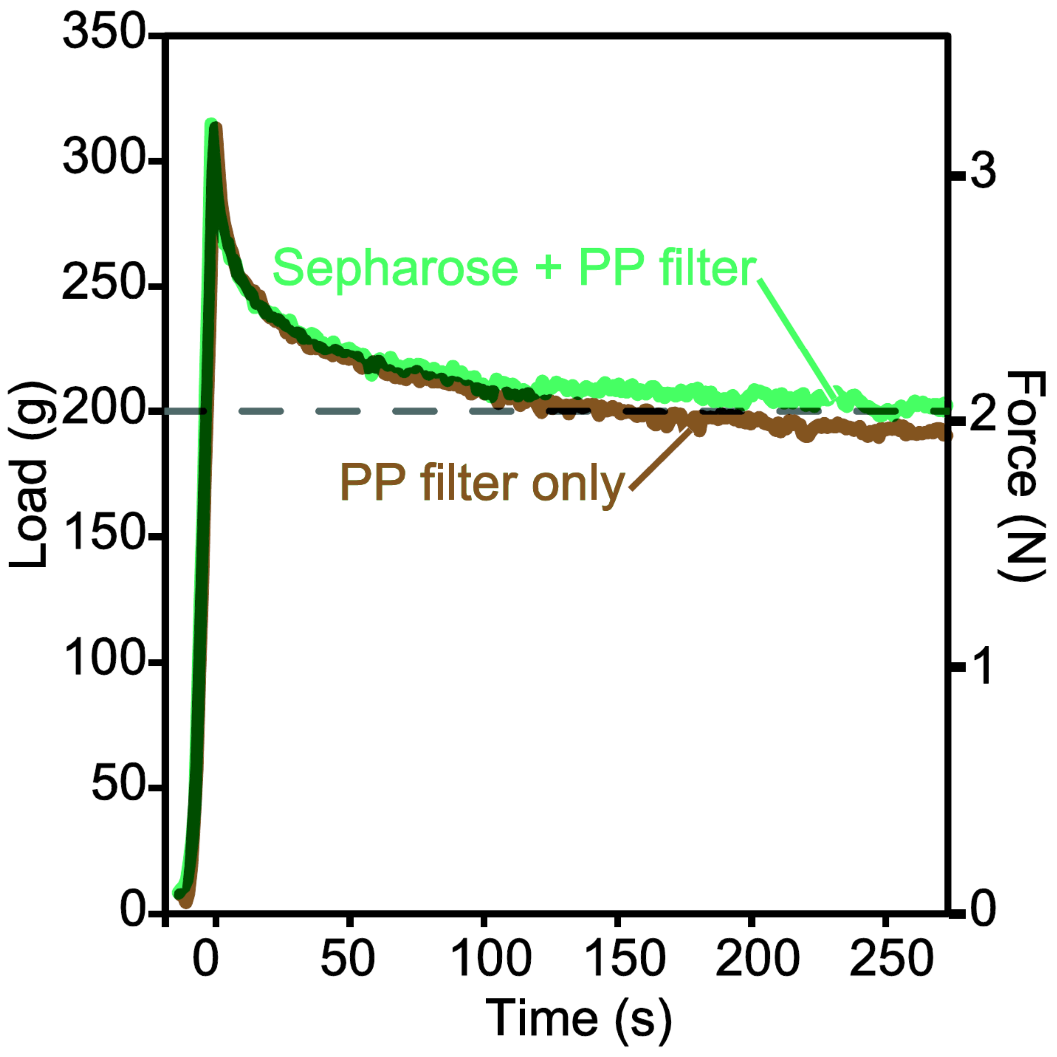

Fig. S1 Load measured by the load cell following the lowering of the plunger

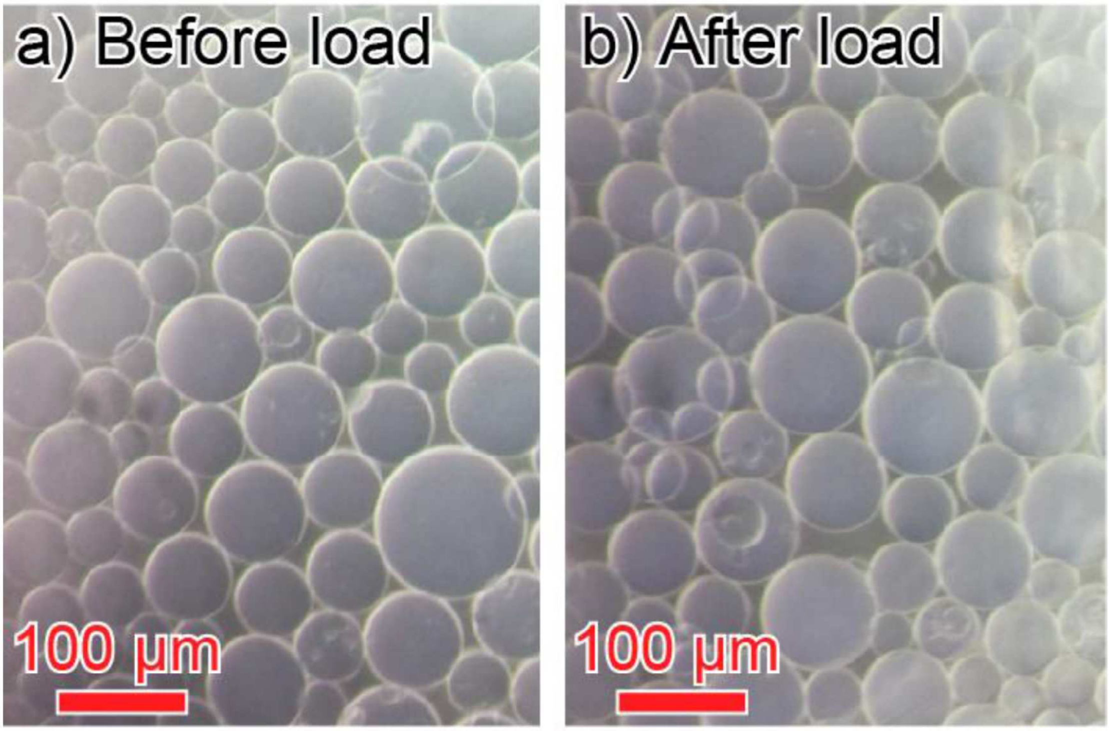

Fig. S2 Microscopy image of resin beads following ATR-FTIR spectroscopic measurements

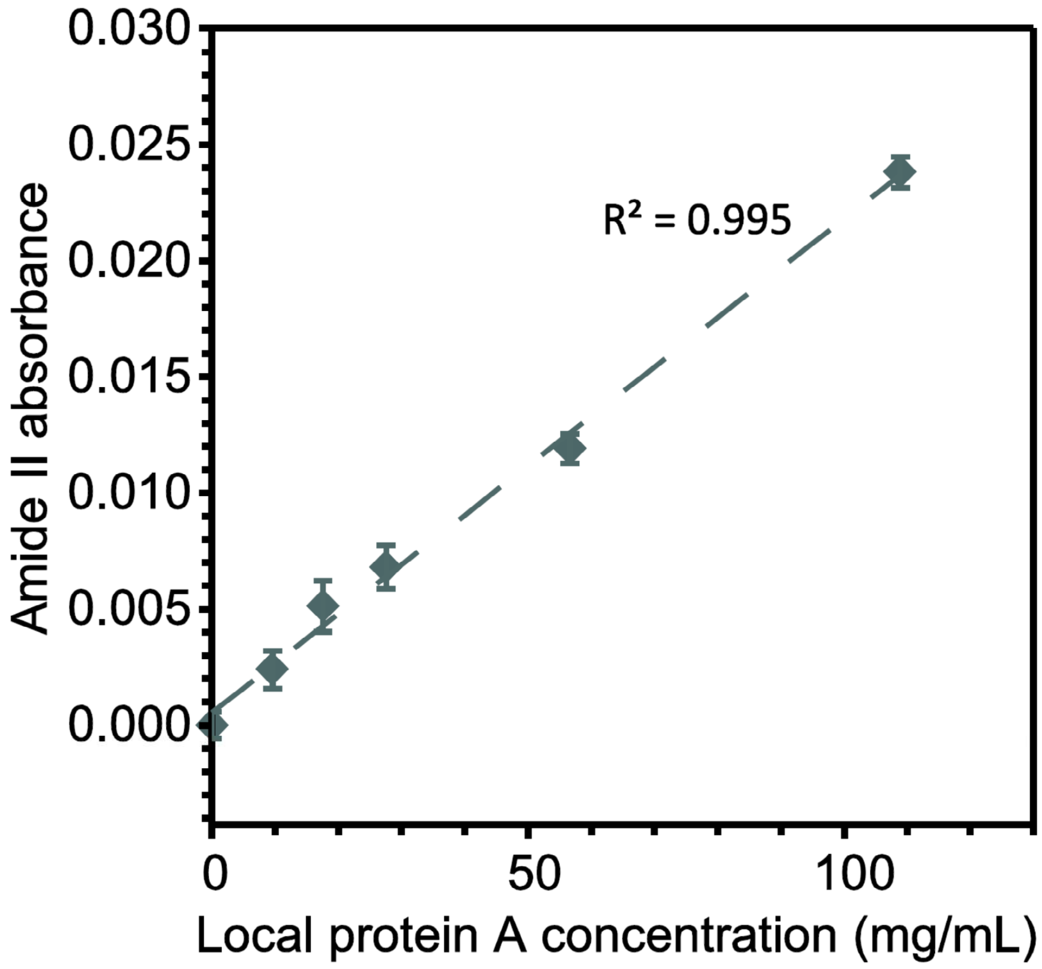

Fig. S3 Standard addition curve used for Protein A local concentration quantification

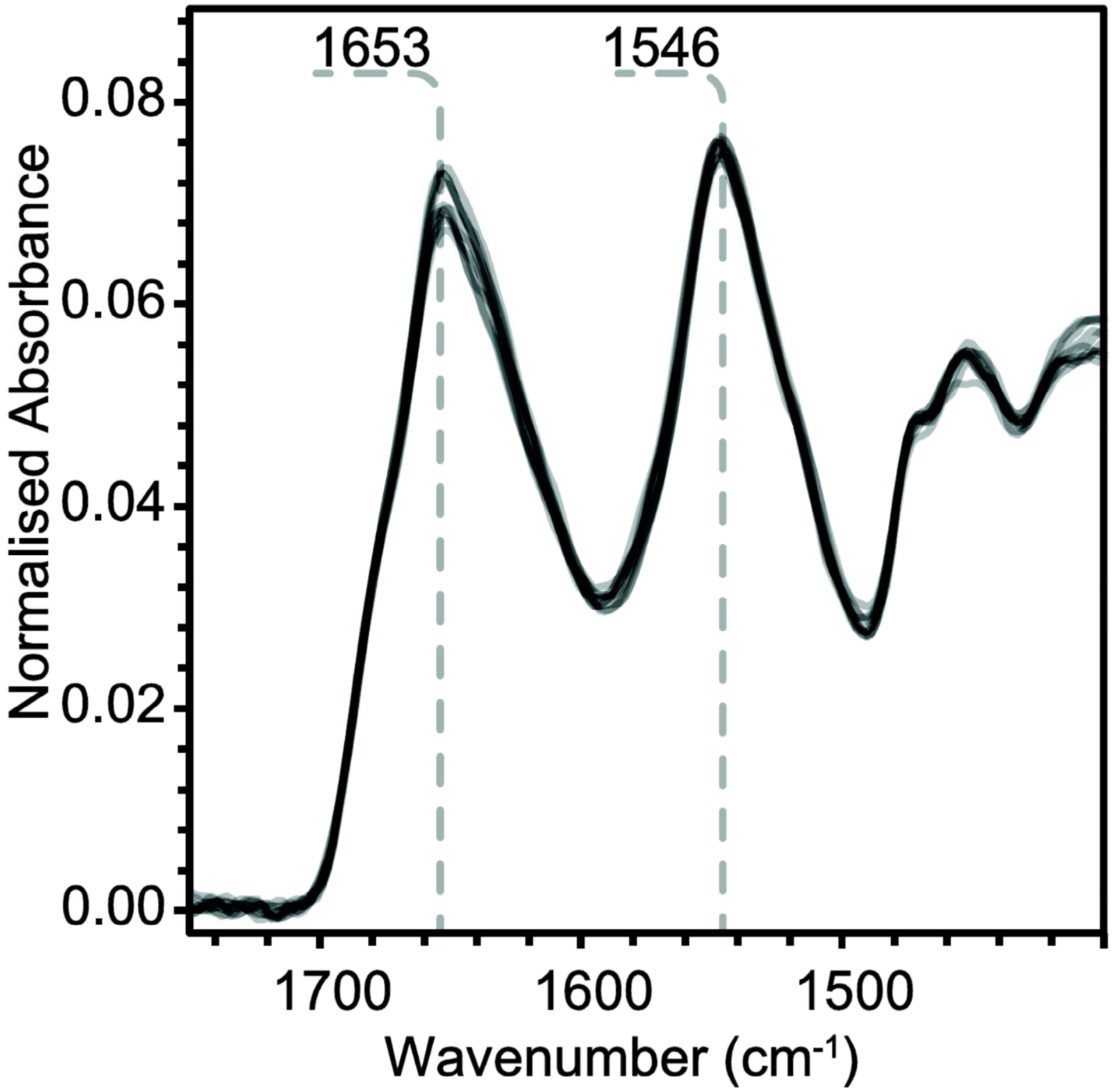

Fig. S4 Spectral region for Amide I and II bands of 10 different rProtein A Sepharose resin aliquots measured by ATR-FTIR spectroscopy under 200 g load. Spectra were normalized using the 1050 cm^{-1} polysaccharide band. The overlapping spectra are indicative of the loading reproducibly
